# Spectroscopic investigation of size-dependent CO_2_ binding on cationic copper clusters: analysis of the CO_2_ asymmetric stretch†

**DOI:** 10.1039/d4cp01797h

**Published:** 2024-07-12

**Authors:** A. M. Reider, M. Szalay, J. Reichegger, J. Barabás, M. Schmidt, M. Kappe, T. Höltzl, P. Scheier, O. V. Lushchikova

**Affiliations:** a Institut für Ionenphysik und Angewandte Physik, Universität Innsbruck Technikerstraße 25 Innsbruck 6020 Austria olga.lushchikova@uibk.ac.at; b HUN-REN-BME Computation Driven Chemistry Research Group, Department of Inorganic and Analytical Chemistry, Budapest University of Technology and Economics Muegyetem rkp. 3 Budapest 1111 Hungary; c Furukawa Electric Institute of Technology Késmárk Utca 28/A Budapest 1158 Hungary

## Abstract

Photofragmentation spectroscopy, combined with quantum chemical computations, was employed to investigate the position of the asymmetric CO_2_ stretch in cold, He-tagged Cu_*n*_[CO_2_]^+^ (*n* = 1–10) and Cu_*n*_[CO_2_][H_2_O]^+^ (*n* = 1–7) complexes. A blue shift in the band position was observed compared to the free CO_2_ molecule for Cu_*n*_[CO_2_]^+^ complexes. Furthermore, this shift was found to exhibit a notable dependence on cluster size, progressively redshifting with increasing cluster size. The computations revealed that the CO_2_ binding energy is the highest for Cu^+^ and continuously decreases with increasing cluster size. This dependency could be explained by highlighting the role of polarization in electronic structure, according to energy decomposition analysis. The introduction of water to this complex amplified the redshift of the asymmetric stretch, showing a similar dependency on the cluster size as observed for Cu_*n*_[CO_2_]^+^ complexes.

## Abstract 2

The asymmetric stretch vibration of CO_2_ on different Cu_*n*_^+^ cluster sizes, with *n* = 1–10, is spectroscopically investigated. The strongest binding is found with Cu^+^, decreasing with cluster size, as reflected in the vibration frequency shift.

## Graphical abstract

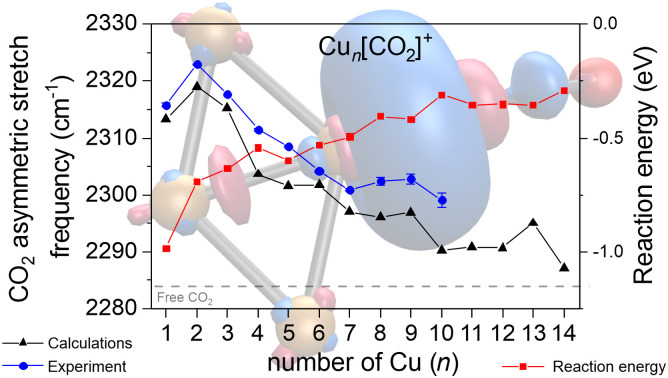

## Introduction

The increasing concentration of atmospheric carbon dioxide has become a pressing global concern due to its implications for climate change and environmental sustainability. However, its conversion into valuable chemicals remains a challenging process characterized by low CO_2_ conversion rates, poor selectivity, and high energy costs.^1,2^

One of the most promising pathways for CO_2_ utilization is its conversion to methanol. Behrens *et al.* demonstrated that Cu nanoparticles serve as the active sites in the widely used industrial Cu/ZnO/Al_2_O_3_ catalyst, which has been on the market for over six decades.^3^ Recent reviews corroborate that Cu-based catalysts continue to show the greatest promise.^4,5^ The emergence of small sub-nanometer-sized metal clusters exhibit a high potential in sustainable CO_2_ conversion reactions due to their high number of uncoordinated sites and distinctive electronic and geometric properties, which can facilitate the activation and transformation of CO_2_ molecules.^6^ Over the past decade, copper clusters have attracted considerable attention for their potential to convert CO_2_ to methanol at lower temperatures and pressures compared to conventional catalysts.^7–12^

It is widely acknowledged that the size of a cluster can significantly influence its catalytic properties, potentially enhancing the activity of the catalyst. The active sites of catalysts can be imitated by gas-phase clusters to investigate the exact reaction mechanisms to obtain detailed knowledge of this phenomenon.^13^ Gas-phase studies offer the advantage of examining the interaction between CO_2_ and a cluster under well-controlled conditions. Over the years, substantial knowledge has been accumulated regarding the interaction between gas-phase ions and CO_2_, as comprehensively summarized by H. Schwarz.^14^

Spectroscopic studies, in particular, provide detailed information about the vibrational properties of adsorbed molecules, shedding light on the nature of the adsorption process and the strength of the metal–ligand bonds. Several research groups have employed IR photodissociation spectroscopy techniques, such as eliminating one or more intact CO_2_ molecules or employing Ar tagging for tightly bound complexes. Notably, Weber^15–25^ and his co-workers extensively explored the interaction of single metal anions with multiple CO_2_ molecules, while the groups of Duncan,^26–32^ Mackenzie,^33,34^ and Jiang^35–38^ focused on cations and their oxides. Their works cover the interactions of CO_2_ with most transition metal ions and beyond. Recently, the group of Beyer has published spectroscopic studies on the activation of CO_2_ by hydrated metal cations (Mg^+^ and Co^+^)^39,40^ and anions (NbO_3_^−^)^41^ solvated in water, revealing that a higher water load can lead to the activation of CO_2_ inducing charge transfer from the metal center to CO_2_.

Other spectroscopic investigations focusing mainly on the interactions of metal clusters with only a few CO_2_ molecules attached employed infrared multiphoton dissociation (IRMPD) spectroscopy with the free-electron lasers (FEL) at the Fritz Haber Institute (FHI) in Berlin, Germany, and at FELIX in Nijmegen, the Netherlands. While at the FHI the focus was more on anionic clusters (Pt_*n*_^−^ and Co_*n*_^−^),^42,43^ the research at FELIX was mostly leaning towards cationic clusters (Cu_*n*_^+^ and Mg_*n*_O_*m*_^+^),^44,45^ with the exception of CCu_*n*_^−^.^46^ In addition, the activation of H_2_O and CO_2_ by NbO_3_^−^ clusters was investigated at FELIX.^41^ Remarkably, all investigated anionic metal clusters exhibited pronounced size-dependent activation and dissociation of CO_2_.^42,43,46^

While it is evident that anions exhibit greater activity in CO_2_ dissociation due to their ability to donate an electron to the anti-bonding orbitals of CO_2_, surface studies highlight the crucial role of Cu particles with a positive partial charge in methanol synthesis.^11,47,48^ These particles stabilize reaction intermediates, promote hydrogenation reactions by facilitating the adsorption and activation of hydrogen molecules, and exhibit enhanced catalytic activity. This underscores the importance of understanding the reaction mechanism over positively charged metal particles. The research of Bakker's group, conducted at FELIX, delved into the elementary steps of methanol formation utilizing cationic Cu clusters, including hydrogenation of the clusters^49^ and CO_2_ and H_2_ co-adsorption.^44^ Those studies revealed that some cluster sizes have the capability to dissociate H_2_, and the co-adsorption studies suggest the formation of intermediates leading to the subsequent formation of formate—a critical step in methanol synthesis. Intriguingly, the reaction of bare cationic Cu clusters with CO_2_ revealed that clusters ranging from 7 to 25 Cu atoms physisorbed CO_2_ uniformly in monodentate (η^1^-O) fashion, regardless of the cluster size, as evidenced by absorption bands measured close to those of free CO_2_ and calculations.^44^

The measurements described above do not really capture the strongest fundamental vibration of CO_2_, the asymmetric stretch (ν_3_), since they were limited by the frequency capabilities of the FEL for intracavity experiments (FELICE) used for those studies, which is 100–2100 cm^−1^. The asymmetric stretch is only captured by the second harmonics of IR-light at 1185 cm^−1^, halving the frequency, and consequently reducing intensity to approximately 1%.^44^

In this work we employ photodissociation spectroscopy of He-tagged Cu_*n*_[CO_2_]_*m*_^+^ complexes, facilitated by a table-top OPO laser, to investigate the strongest vibration of CO_2_, the asymmetric stretch. He-tagged ions are generated and extracted from multiply-charged superfluid He nanodroplets (HNDs).^50^ The narrower spectral bandwidth of the OPO laser compared to that of FELICE, lower dissociation energy of He compared to CO_2_ (<0.04 eV *vs.* >0.26 eV) and low temperature of the formed complexes (<1 K) contribute to the narrower bandwidth, making it possible to investigate the exact position of the ν_3_ band, which is expected around 2350 cm^−1^, according to both studies at FELIX^44^ and data obtained for free CO_2_.^51^

Previously, HNDs were already utilized to study the reactions of neutral metal–CO_2_ complexes. This method employs optically selected mass spectrometry with a narrow bandwidth laser, resulting in high-resolution IR spectra. It was observed that both studied metal atoms, Al and Mn, form two isomers with CO_2_. This conclusion was based on the presence of two bands around 2280 cm^−1^, assigned to the different asymmetric stretches of different isomers. The CO_2_ was found bound to the metal atom in either a monodentate or T-shaped configuration.^52^

By employing IR spectroscopy of He-tagged ions coupled with theoretical calculations, we systematically investigate the influence of cluster size and hydration on the binding motifs of CO_2_ characterized by the changes in the asymmetric stretch mode. This approach allows discerning size-dependent trends in CO_2_ adsorption on copper clusters, the effect of the additional water attachment, and correlating these to the changes in the binding energies. It sheds light on the underlying mechanisms governing the interaction between CO_2_ molecules and metal clusters.

The narrow bandwidth allows for tracking the shift of the ν_3_ band as a function of cluster size, which had not been observed previously. In earlier measurements, all investigated cluster sizes (*n* = 4–25) exhibited identical behavior, with all bands at the same position.^44^ High-level calculations accurately predicted this red shift and found a strong correlation with the reaction energy and polarization. The current work also includes clusters ranging in size from one to three atoms and investigates the influence of hydration on the interaction with CO_2_, areas that had not been studied before. The results show that the monoatomic Cu cation has the highest reaction energy with CO_2_, which decreases with increasing cluster size in both pristine and hydrated clusters.

## Methods

### Experimental

This section provides an overview of the setup of ClusTOF, adapted to the presented experiment. More detailed insights into the formation, ionization and doping processes of superfluid helium droplets in the apparatus are given in the literature.^53,54^

**Fig. 1:**
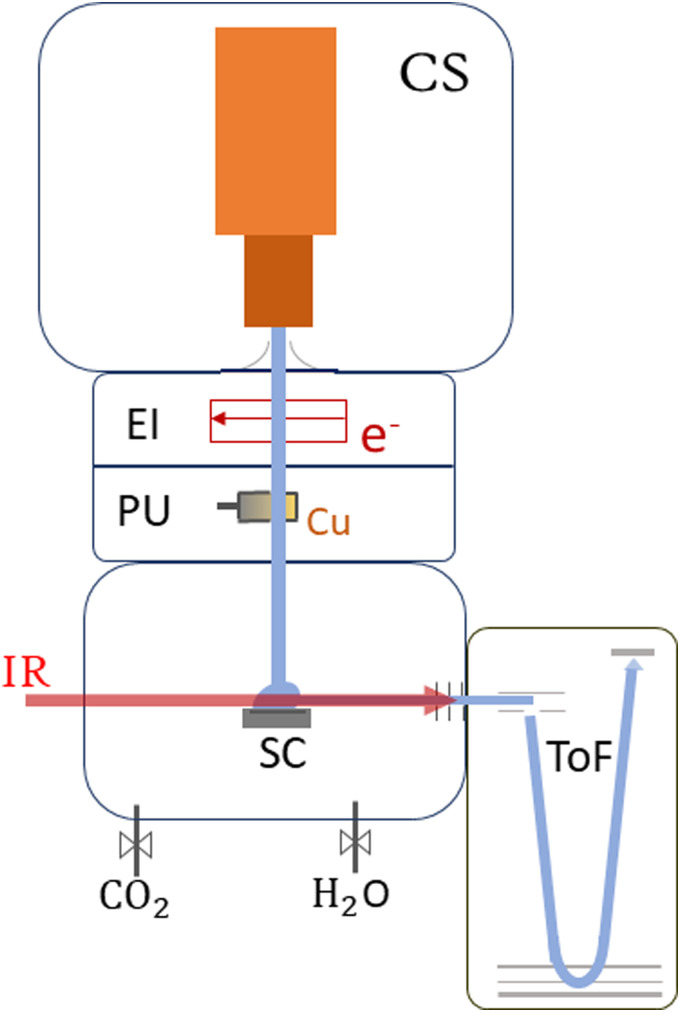
Schematic sketch of the ClusTOF experiment, more details can be found in the text. HNDs are produced by the cluster source (CS). After multiply charging the droplets *via* electron impact (EI), they are doped with evaporated copper in the pick-up chamber (PU), followed by the pick-up of CO_2_ and, if desired, H_2_O. After cluster formation, the HNDs collide with a surface (SC), liberating He-tagged dopants feasible for analysis and detection with the time-of-flight (ToF) mass spectrometer. Action spectroscopy is achieved by irradiating the extracted cluster ions with an IR laser before entering the ToF.

The experimental process, see Fig. 1, is initiated by the formation of HNDs, several 10^5^ helium atoms,^55^ when pre-cooled (9.4–9.7 K) and pressurized (20–22 bar, purity 99.9999%, Messer) helium is expanded through a pinhole nozzle, with the diameter around 5 μm, into the ultra-high vacuum. The temperature and pressure of the helium as well as the current and energy of the ionizing electron beam are finely controlled to ultimately optimize the ion yield of helium-tagged Cu_*n*_[CO_2_]^+^ or Cu_*n*_[CO_2_][H_2_O]^+^ clusters. The HNDs are ionized *via* electron impact to create multiply-charged droplets, resulting in this experiment in a charge state around +10*e*.^56^ In two consecutive, differentially pumped vacuum chambers, the droplets then pick up any species brought into the gas phase, explicitly copper atoms (isotopically enriched ^63^Cu, isotope Rosatom 99.8% purity; evaporated in a home-built, resistively heated ceramic oven), CO_2_ (isotopically enriched ^13^C^16^O_2_, Sigma Aldrich purity 99.0%) and H_2_O (both introduced *via* separate gas inlets with needle valves). This pickup of dopants in combination with charge transfer from He_*n*_^+^ ionic cores leads to the formation of singly-charged dopant ions^57^ and attachment of further dopants results in cluster ions^58^ in the form of Cu_*n*_[CO_2_]_*m*_^+^ and Cu_*n*_[CO_2_]_*m*_[H_2_O]_*k*_^+^. The temperature of the oven as well as the partial pressures of water and carbon dioxide are tuned to optimize the ion yield of copper cluster ions complexed with one CO_2_ or one CO_2_ plus one H_2_O molecule. The charged droplets then collide with a surface,^59^ liberating cold, helium-tagged ions, which are analyzed using a time-of-flight mass spectrometer combined with infrared IR photon absorption from a tunable OPO laser (EKSPLA NT277, linewidth ∼8 cm^−1^). In this form of action spectroscopy, the absorption of a photon leads to the loss of He atoms and enables the determination of absorption spectra *via* the increase of the photoproducts.^50^ The mass spectrometer runs at a 10 kHz extraction rate, while the laser operates at 1 kHz. This ensures that every tenth extraction pulse coincides with laser illumination, facilitating side-by-side comparison of mass spectra with and without laser interaction. Absorption of a resonant photon leads to evaporation of the weakly bound He tags, observed in the mass spectrum as a depletion of the Cu_*n*_[CO_2_]_*m*_[He]_*x*_^+^ ion signal and the simultaneous increase of the photoproduct, the bare Cu_*n*_[CO_2_]_*m*_^+^ ion. The resulting ion yield is then defined as the ratio of the signal of the bare ion with and without laser exposure. All spectral results are corrected for background noise by subtracting the signal of IR-inactive ions from the IR-active ions at each ToF extraction. No power correction has been employed since it remains flat in the region where the complexes absorb the radiation. The laser power at each scanned wavelength can be found in Fig. S1 in the ESI.†

### Computational

Density functional computations were carried out using the Q-Chem 6.1 program package^60^ to recompute previously reported most stable Cu_*n*_^+^ (*n* = 1–14) cluster geometries.^61,62^ Here the pre-screened possible reaction products, both with intact CO_2_ (Cu_*n*_[CO_2_]^+^); with intact CO_2_ and intact H_2_O (Cu_*n*_[CO_2_][H_2_O]^+^); and with intact CO_2_ and H_2_O, dissociated to H and OH (Cu_*n*_[CO_2_][H][OH]^+^) are reported using the PBE/LANL2DZ level of theory.

The in-house code is utilized to systematically generate the initial geometries for different binding modes, similar to that in our previous works.^46,63,64^ The CO_2_ is bound to the Cu_*n*_^+^ (*n* = 1–14) clusters in mono-(η^1^-O) or bidentate (η^2^-C, O) modes. H_2_O is bound to the most stable Cu_*n*_[CO_2_]^+^ structures for *n* = 1–10. The monodentate mode (η^1^-H) was selected for intact H_2_O, while H was bound in bridge mode over adjacent coppers, or over a three-membered copper ring, and OH was bound in bridge mode over adjacent copper atoms or was bound to a single copper, as the water molecule may dissociate on copper clusters.^65,66^ Please note that the goal is not to locate the global minimum structures, but rather to locate the possible products, accessible by the addition of the reactants to the cluster. All isomers are relaxed and calculated with the lowest spin state (*i.e.* singlet or doublet for structures having an even or odd number of electrons, respectively).

Final geometry optimizations and vibrational frequency computations are performed using the (U)TPSSh/def2-TZVP+D3 level of theory. This method has been used previously for similar systems and yielded accurate results compared both to computational benchmarks (up to CCSD(T)/def2-QZVPPD) and to experimental results.^44,46,67^ After the final optimization, the energetic order of the different isomers remained the same as in the pre-screening. The harmonic frequency computations reported in the main text were completed using isotopically pure ^13^C.

The impact of the correlation, basis sets, dispersion correction, relativity and anharmonicity on the computed vibrational spectrum is carefully benchmarked to ensure the most accurate results. The results of benchmarking are shown in Fig. S2 of the ESI.† Anharmonicity calculations shown in Tables S1 and S2 (ESI†) for Cu_*n*_[CO_2_]^+^, with *n* = 0, suggest a scaling factor of 0.98, which is subsequently applied to the results presented in the text. It is worth noting, however, that benchmarking is done for CO_2_ with the ^12^C isotope, while frequencies used for the comparison with the experimental data were corrected for the ^13^C isotope. Binding energies are determined by calculating the difference between the total energy of the complex and the sum of the individual gas phase reactants namely Cu_*n*_^+^, CO_2_, and intact H_2_O.

## Results and discussion

### Spectroscopic investigation

**Fig. 2:**
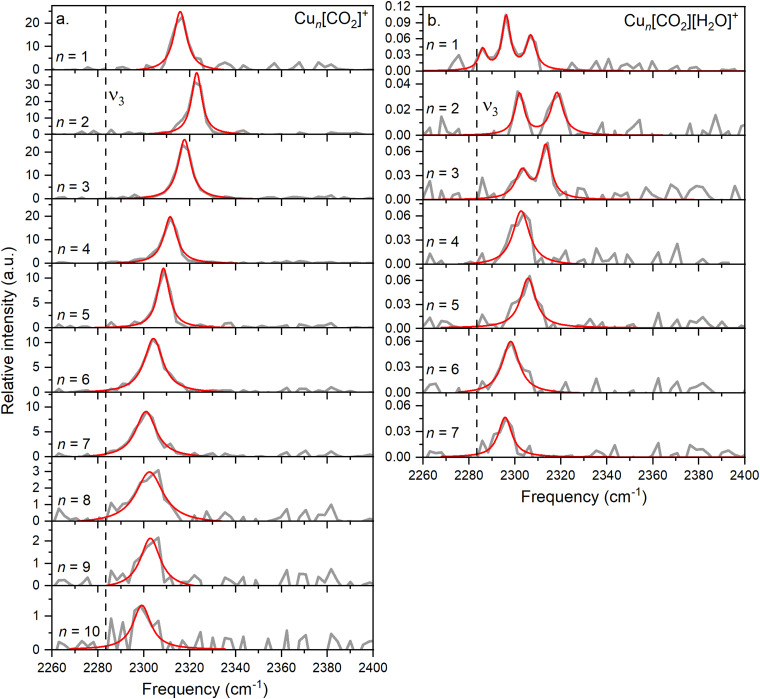
(a) The average of experimental photodissociation spectra of the Cu_*n*_[CO_2_]^+^ complexes, covering the range of *n* = 1–10, within the vibration spectrum of the CO_2_ asymmetric stretch, is showcased in grey. (b) The same spectral range investigated for Cu_*n*_[CO_2_][H_2_O]^+^ complexes, *n* = 1–7. On both graphs, the Lorentzian-fitted bands are highlighted in red. Additionally, the position of the band determined for free CO_2_ is indicated with a dashed line.

In this study, we investigate He-tagged Cu_*n*_[CO_2_]^+^ complexes (*n* = 1–10) formed within helium nanodroplets (HNDs) using photodissociation spectroscopy. Upon photon absorption, the weakly bound helium atoms evaporate, leading to an increase in the ion intensity of the naked complex. To ensure sufficient helium attachment, over 50 helium atoms were attached to each complex, subsequently decaying into the naked complex. The resulting spectra are presented in Fig. 2a.

Based on previous measurements, it is anticipated that CO_2_ will be physisorbed onto the cationic Cu cluster ions.^44^ Consequently, the asymmetric stretch vibration is expected to closely mirror that of free ^13^CO_2_, which has been determined *via* IR spectroscopy in a gaseous phase at 2283.48 cm^−1^, a reference point we will utilize throughout this text.^51^ It is important to note that this experiment was conducted using isotopically enriched CO_2_, with ^13^C, rather than the naturally more abundant one with ^12^C, which would exhibit its asymmetric stretch at 2349.16 cm^−1^.^51^ This isotope was chosen to eliminate possible overlapping of the He-tagging series with the Cu_*n*_[CO_2_]^+^ series in the mass spectrum. The current experiment on the complexes with *n* = 1–10 exhibits a blue shift of at least 15 cm^−1^ compared to the vibration of free CO_2_, falling within the range of 2290–2330 cm^−1^ with an average full width at half maximum (FWHM) of 9 cm^−1^. The signal-to-noise ratio (S/N) decreases with increasing cluster size due to reduced helium attachment for larger clusters, as illustrated in the mass spectrum shown in Fig. S3 (ESI†).

When comparing the interaction of the Cu^+^ monomer with CO_2_ (with the ν_3_ band at 2316 cm^−1^) to monomers of other previously studied metals, first a correction for the heavier isotope of CO_2_ must be made. The reduced mass for naturally abundant ^12^CO_2_ and ^63^Cu is 25.9, while for the heavier ^13^CO_2_ isotope, it is 26.25. Therefore, to compare the results with other experiments, the experimentally obtained frequency is multiplied by a factor of 1.014, resulting in a frequency of 2347 cm^−1^.

Most metal and metal oxide ions investigated to date (including Ni, Si, Ti, Fe, Mg, Co, Rh, Ir, Al, NbO, and TaO) exhibit a band between 2265 and 2385 cm^−1^, which is blue-shifted relative to the asymmetric stretch of free CO_2_, similarly to the one found in the current experiment.^27–31,33,34,68^ When these ions are further solvated with CO_2_ molecules, this band generally shifts further towards the red, closer to the vibrational frequency of free CO_2_, indicating a lower binding energy of subsequent CO_2_ molecules. It's noteworthy that for most of these metals, no data on complexes with only one CO_2_ molecule is reported, hence the band position of two CO_2_ is utilized. Additionally, Ar is used as a tag, as the dissociation of CO_2_ requires higher energy, leading to significant band broadening.^27^

Other metals such as Ca, Sr, Ba exhibit a band around 2245 cm^−1^.^69^ For all these alkaline earth metals, it has been observed that the first CO_2_ molecule is weakly bound in an η^1^ configuration dominated by charge–quadrupole interaction. However, further solvation leads to the blue shift of ν_3_ by at least 10 cm^−1^. From the calculations it becomes evident that the second CO_2_ binds in a bidentate manner due to the transfer of positive charge to the metal core, rendering CO_2_ electronegative and stimulating its activation. A similar effect has also been noted for the solvation of metal–CO_2_ complexes in water.^40,41^

In the current study, the primary focus lies on cluster size rather than solvation effects. The position of the ν_3_ band exhibits a correlation with cluster size, progressively approaching that of free CO_2_ as the cluster size increases, which was not observed before. Drawing an analogy with previous experiments involving ion solvation in multiple CO_2_ molecules, a similar trend is observed, indicating a decrease in reaction energy.

A similar behavior of the CO_2_ asymmetric stretch is observed when H_2_O is co-adsorbed on the cluster. However, it is further redshifted compared to the ν_3_ band of Cu_*n*_[CO_2_]^+^ complexes, as can be seen in Fig. 2b.

A triple band is observed for *n* = 1, which might result from the low S/N. However, based on the width of the peaks and their height, it is highly unlikely that they originate from a single band, as this feature would be much broader than the corresponding bands in the other cluster sizes. Similarly, the spectra for *n* = 2 and *n* = 3 exhibit two bands.

### Computational results

**Fig. 3:**
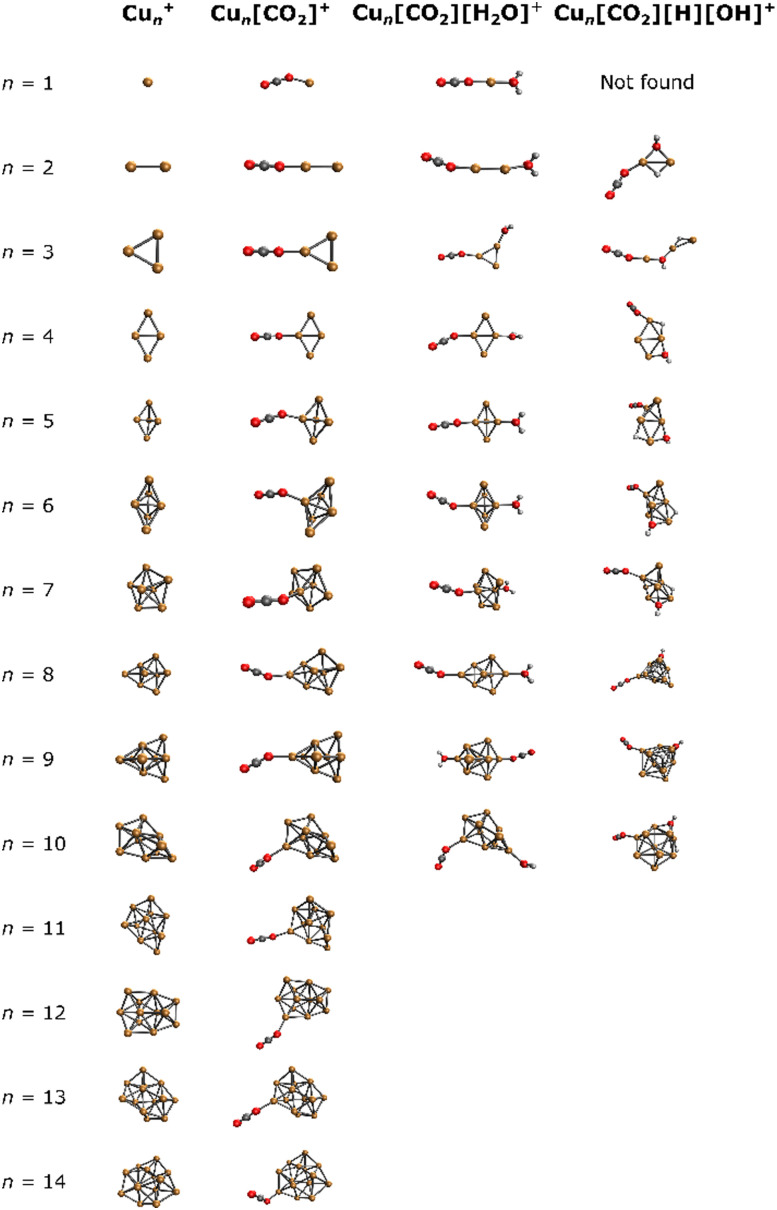
Lowest energy isomers for Cu_*n*_^+^, Cu_*n*_[CO_2_]^+^, Cu_*n*_[CO_2_][H_2_O]^+^, and Cu_*n*_[CO_2_][H][OH]^+^ in the studied binding modes.

For a comprehensive understanding of the investigated complexes, the lowest energy isomers of bare Cu_*n*_^+^ were explored computationally, along with the corresponding lowest energy Cu_*n*_[CO_2_]^+^, Cu_*n*_[CO_2_][H_2_O]^+^ and Cu_*n*_[CO_2_][H][OH]^+^ adducts. The resulting structures are depicted in Fig. 3.

In all Cu_*n*_[CO_2_]^+^ adducts, the CO_2_ molecule binds in an η^1^ configuration with one oxygen to one of the copper atoms at the cluster edge. This binding mode, together with the previous results,^44,46,67^ suggests a barrierless reaction of CO_2_ with the cluster. Upon addition of water, cluster backbone rearrangement was observed only in the case of *n* = 9. However, following H_2_O dissociation, significant cluster geometry changes were observed in almost all cases (except for *n* = 2, 4, 6), indicating a multi-step reaction mechanism. Notably, for *n* = 1, no structure with dissociated H_2_O was found among the selected binding modes.

**Fig. 4:**
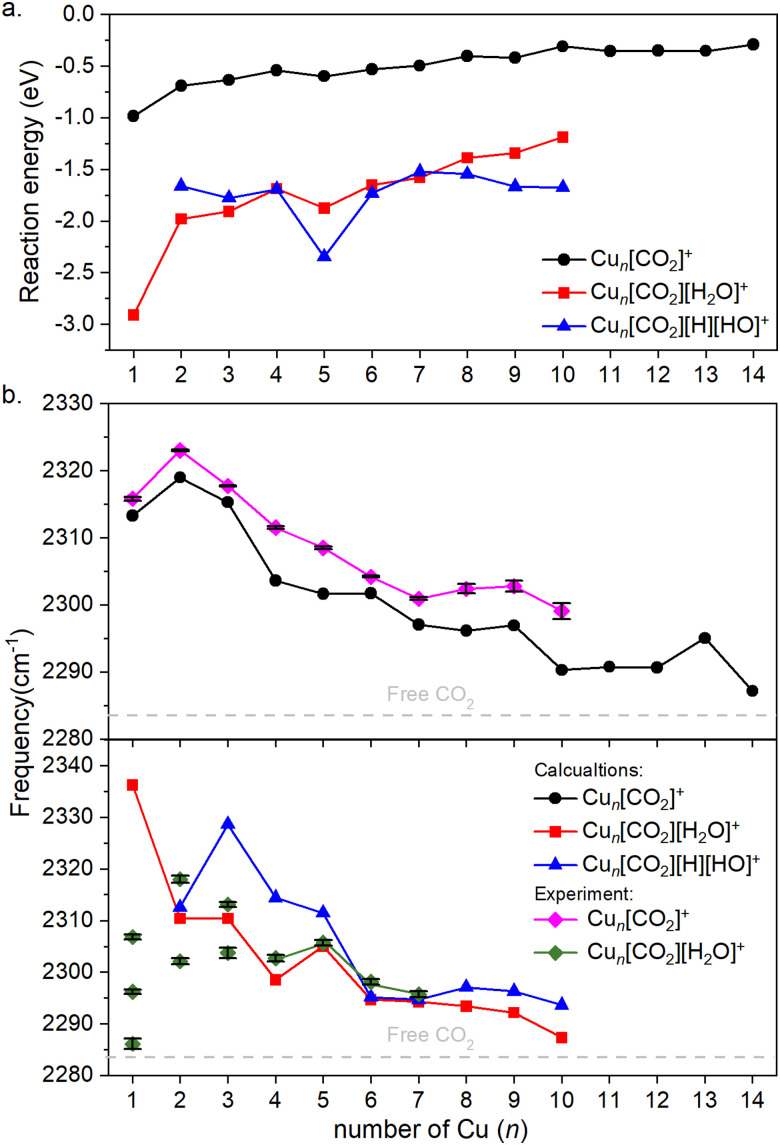
(a) Binding energies of the cluster adducts depicted on Fig. 3. (b) Top: Frequencies of the asymmetric stretching vibrational mode of the bound CO_2_ on corresponding adducts are depicted. Bottom: The frequency of the same vibration, when water is co-adsorbed. The diamonds denote the experimental results, which include error bars. Note that the error bars are often smaller than the symbols; therefore, a black color is used to represent them. Additionally, for Cu_*n*_[CO_2_][H_2_O]^+^, for *n* = 1–3 the symbols are not connected, since it is not clear which of them might originate from the ν_3_ vibration. The dashed grey line represents the vibration of free CO_2_.

Fig. 4a illustrates the binding energies of the aforementioned structures. In all cases, both CO_2_ and subsequent water adsorption are thermodynamically favorable. However, during the water dissociation, significant stabilization was observed only in the case of *n* = 5, 9 and 10, suggesting that water dissociation is less probable for other cluster sizes.

For both the CO_2_ adsorption and its co-adsorption with water, the CO_2_ binding becomes less favored with increasing cluster size. This trend is also reflected in the frequencies of the asymmetric stretching vibrational mode of adsorbed CO_2_, which converge toward that of free CO_2_, as shown in Fig. 4b.

The overall trend is in good agreement with the calculations, as evidenced by recognizable dips at *n* = 1, 4, and 7 for both experimental data and theoretical predictions for Cu_*n*_[CO_2_]^+^ in the top of Fig. 4b. However, discrepancies arise when comparing co-adsorption with water in the bottom of Fig. 4b: while the calculations suggest no double or triple peaks, they are experimentally observed for *n* = 1–3. Notably, the peak with the highest measured frequency for *n* = 1 is almost 40 cm^−1^ lower, than what was calculated for Cu[CO_2_][H_2_O]^+^. For *n* = 2, the calculated asymmetric CO_2_ stretching frequency in Cu_2_[CO_2_][H_2_O]^+^ and Cu_2_[CO_2_][H][OH]^+^ are remarkably similar to each other as well as to the average of the two measured peaks. In contrast, for *n* = 3, the calculated frequencies for intact and dissociated water are 20 cm^−1^ apart, with the frequency of Cu_3_[CO_2_][H_2_O]^+^ falling between the experimentally determined peaks. All following cluster sizes exhibit only one band, which is in good agreement with the calculations.

According to the computations, the shift in the asymmetric stretch vibrational frequency of the bound CO_2_ due to the H_2_O adsorption is generally low, although somewhat larger in smaller sizes (*n* = 1–4), since CO_2_ and H_2_O are bound to the same (*n* = 1) or adjacent copper atoms (*n* = 2–4). A good correlation between the vibrational frequencies and the binding energies for Cu_*n*_[CO_2_]^+^ (*R*^2^ = 0.743) and Cu_*n*_[CO_2_][H_2_O]^+^ (*R*^2^ = 0.963) is observed as evident from Fig. S4 (ESI†). However, it is much lower for Cu_*n*_[CO_2_][H][OH]^+^, with *R*^2^ = 0.141. Accordingly, in the case of Cu_*n*_[CO_2_][H_2_O]^+^ the binding energy is significantly more negative for *n* = 1, thus the CO_2_ asymmetric stretching vibrational frequency is considerably increased in the next largest cluster.

**Fig. 5:**
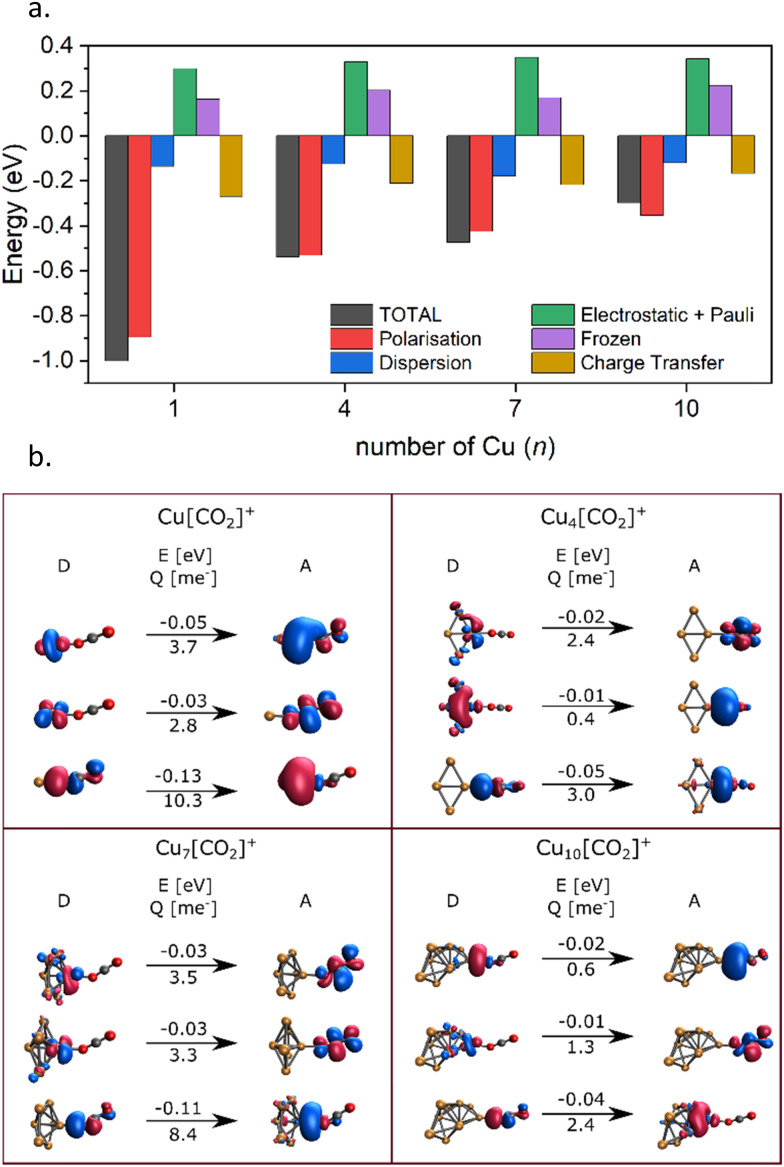
(a) Energy decomposition analysis for Cu_*n*_[CO_2_]^+^ adducts (*n* = 1, 4, 7, 10). (b) Major complementary occupied-virtual pairs (COVP) and their contribution to the charge transfer energy changes (above the arrow in kJ mol^−1^) and transferred charge (under the arrow in me-) (D = donor, A = acceptor).

**Fig. 6:**
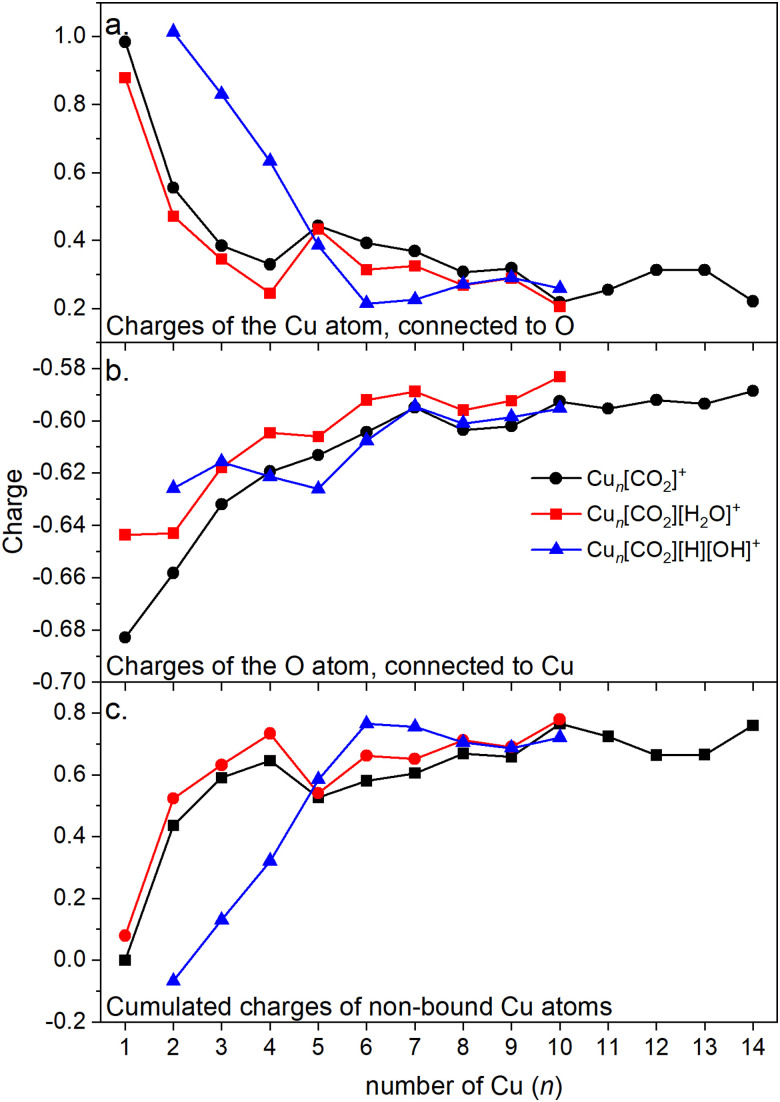
(a) Charges of the Cu atom which is connected to the CO_2_ in Cu_*n*_[CO_2_]^+^. (b) Charges of the O atom in Cu_*n*_[CO_2_]^+^ which is connected to the cluster. (c) Cumulated charges of the copper atoms except the Cu atom which is connected to the CO_2_ in Cu_*n*_[CO_2_]^+^ (copper atoms not bound to CO_2_).

**Fig. 7:**
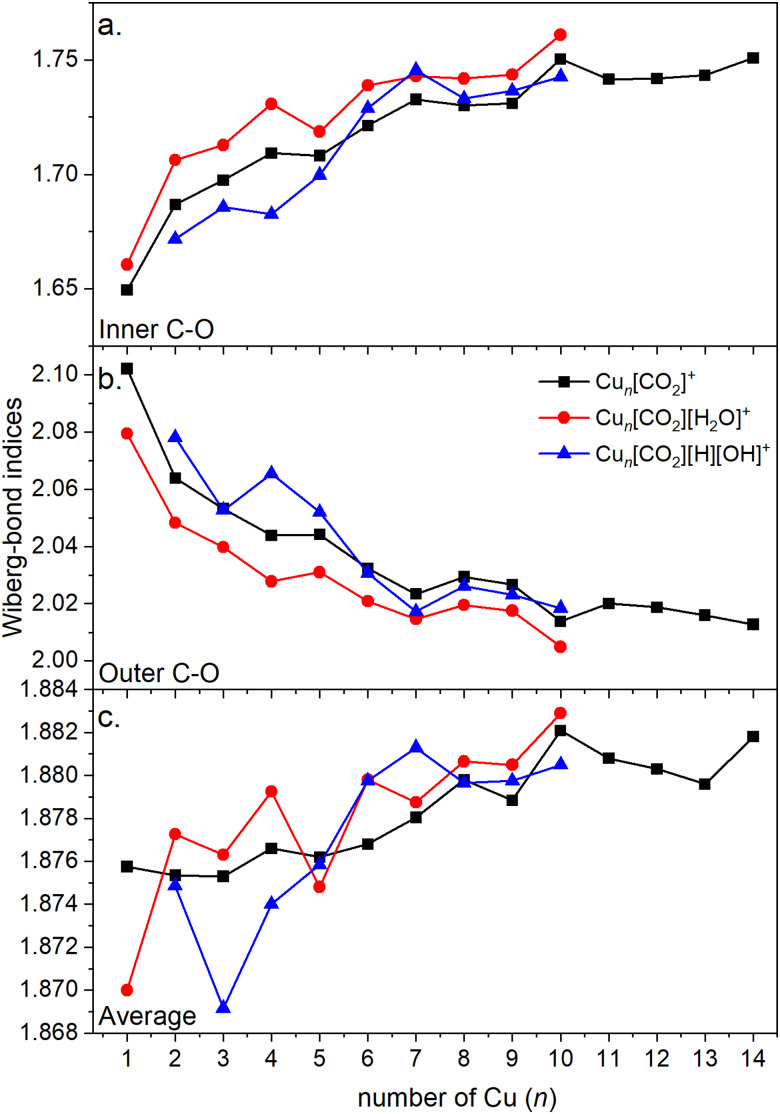
Wiberg bond indices of (a) the C–O bond (where oxygen is connected to the cluster, *i.e.* inner oxygen); (b) the C–O bond (where oxygen is not connected to the cluster, *i.e.* outer oxygen); (c) both C–O bonds (inner and outer) with respect to the cluster size.

Energy decomposition analysis (EDA) was performed for several cluster sizes to gain a better understanding of the molecular interactions and rationalize the reactivity of the clusters. The aim of the EDA is to decompose the interaction energy between two fragments (in this case, the Cu_*n*_^+^ clusters and CO_2_) with an adduct into chemically meaningful terms. The EDA based on absolutely localized molecular orbitals (ALMO-EDA) is employed, where ALMOs are localized on each fragment facilitating a detailed analysis of intermolecular interactions.^70^ ALMO-EDA decomposes the total interaction energy (here, binding energy) into frozen terms encompassing electrostatic interaction, Pauli repulsion, and dispersion interaction between the fragments, along with polarization, charge transfer and relaxation terms. The charge transfer energy can be further decomposed exactly to contributions from complementary occupied-virtual pairs (COVPs), wherein each orbital pair member resides on one of the fragments. Consequently, COVPs visually highlight the most significant orbitals involved in charge transfer. These analyses are illustrated in Fig. 5. Fig. 5a shows the EDA, without the relaxation energies, because these values are practically zero (around 0.01 eV), as expected since no significant geometry changes occur in the fragments due to the CO_2_ adsorption on the clusters. Polarization in the fragments is the main term in the binding energies, which is strongly decreasing with the cluster size. Charge transfer (CT) follows the same trend, although with a significantly lower magnitude. Fig. 5b shows the main complementary occupied-virtual pairs (COVP) based on the values of the energy transfer. Donation from the clusters’ d_*z*^2^_ and d_*zx*_ orbitals to the LUMO of CO_2_ weakens the carbon–oxygen bond. Additionally, back donation occurs, where the lone electron pair of the oxygen atom in CO_2_ donates electron density to the formally unoccupied 4p orbital of the copper atoms, albeit with a somewhat lesser contribution. However, it's crucial to note that low values of both donation and back-donation indicate a lesser significance of the charge transfer process compared to the polarization. The approximately equal values of charge transfer contribute to the charge neutrality of the bound CO_2_, with a total charge of around ∼0.02. On the contrary, polarization exerts a more noticeable effect. Fig. 6a and b show the natural charges of the connected Cu and O atoms, while Fig. 6c shows the total charge of the remaining copper atoms in the cluster. Additionally, charges of C and outside O can be found in Fig. S5 (ESI†). The two fragments, namely the CO_2_ molecule and the Cu_*n*_^+^ cluster, exert a mutual polarization effect. This results in the connecting oxygen atom of the CO_2_ molecule accumulating a more negative charge (by the electron transfer from the other oxygen atom), while the connecting copper atom in the cluster experiences a corresponding increase in positive charge compared to the other copper atoms in the cluster. This effect diminishes with increasing cluster size, as can be seen from Fig. 6a and b, as charges can be more readily redistributed across larger clusters (see Fig. 6c). Consequently, a less pronounced positive charge can be attained on the copper atom connected to the CO_2_. While bond indices (Fig. 7) between the C and inner O are increasing with the cluster size, those between the C and the outer O are decreasing almost to the same extent. The increased O^inner^–C and the decreased O^outer^–C bond orders are in line with the polarization effects, shown already by the energy decomposition analysis, while the slightly decreased average O–C bond orders suggest the effect of a small charge transfer. Interestingly, the cluster size-dependent asymmetric stretch vibrational frequency correlates with both the increased and the decreased C–O bond indices, and also with their average, as can be seen in Fig. S6 (ESI†). As the asymmetric stretch involves both C–O bonds and the distance between the inner oxygen and the connecting copper atom also changes, all these together determine the corresponding vibrational frequency. According to our computations, the asymmetric vibrational stretch frequency correlates with the CO_2_ binding energy, which according to the EDA is determined mainly by polarization.

## Conclusion

In conclusion, our spectroscopic investigation of He-tagged Cu_*n*_[CO_2_]^+^ and Cu_*n*_[CO_2_][H_2_O]^+^ complexes within helium nanodroplets has provided valuable insights into the interaction between metal clusters and CO_2_ molecules. Through photodissociation spectroscopy, we observed a blue shift in the asymmetric stretch vibration of CO_2_ compared to gas phase CO_2_, with this shift diminishing as cluster size increases. This trend indicates a reduction in binding energy with increasing cluster size, a result supported by a computational analysis. Further computational examinations provided detailed insights into the binding energies and structural alterations upon the adsorption of CO_2_ and water. The energy decomposition analysis underscored the significance of polarization over charge transfer in cluster–CO_2_ interactions. Collectively, our findings highlight the intricate interplay among cluster size, molecular structure, and reactivity, offering promising avenues for the rational design of catalytic systems centered around metal clusters.

## Author contributions

A. M. Reider: investigation, visualization, writing – original draft preparation. M. Szalay: investigation, formal analysis, writing – original draft preparation, visualization. J. Reichegger: investigation, formal analysis, visualization, writing – review & editing. J. Barabas: investigation, formal analysis. M. Schmidt: investigation. M. Kappe: investigation, methodology. P. Scheier: conceptualization, funding acquisition, resources, methodology, conceptualization, software, writing – review & editing. T. Höltzl: conceptualization, investigation, formal analysis, visualization, writing – original draft preparation, review & editing. O. V. Lushchikova: conceptualization, data curation, funding acquisition, investigation, project administration, supervision, visualization, writing – original draft preparation.

## Data availability

The data discussed in the manuscript is available at: https://doi.org/10.5281/zenodo.11578007. This repository contains the raw data for the spectroscopic measurements of Cu_*n*_[CO_2_]^+^ (*n* = 1–10) and Cu_*n*_[CO_2_][H_2_O]^+^ (*n* = 1–7) complexes, along with laser power scans. An Excel file is included, summarizing the measurements and the most important experimental parameters. Additionally, we provide the Python script used to extract the IR spectra for the individual cluster sizes, and the Origin file containing the data after laser power corrections, averaging, and the figures used in the original manuscript and the ESI.†

## Conflicts of interest

There are no conflicts to declare.

## Supplementary Material

CP-026-D4CP01797H-s001
