# Supplementary material for: Spectroscopic investigation of size-dependent CO2 binding on cationic copper clusters: analysis of the CO2 asymmetric stretch
Source: Phys Chem Chem Phys. 2024 Jul 12;26(30):20355–64. doi: 10.1039/d4cp01797h (PMC11290062; doi:10.1039/d4cp01797h)
Supplement: CP-026-D4CP01797H-s001 [file CP-026-D4CP01797H-s001.pdf]

This journal is © the Owner Societies 2024

Supporting Information

Spectroscopic Investigation of Size-Dependent CO_2 Binding on Cationic Copper Clusters: Analysis of CO_2 Asymmetric Stretch

A.M. Reider[1], M. Szalay[2,3], J. Reichegger[1], J. Barabas[2,4], M. Schmidt[1], M. Kappe[1], T. Höltzl[2,3]*, P. Scheier[1], O.V. Lushchikova[1]*

[1.] *Institut für Ionenphysik und Angewandte Physik, Universität Innsbruck, Technikerstraße 25, Innsbruck 6020, Austria.*
[2.] *HUN-REN-BME Computation Driven Chemistry Research Group, Department of Inorganic and Analytical Chemistry, Budapest University of Technology and Economics, Muegyetem rkp. 3, Budapest 1111, Hungary*
[3.] *Furukawa Electric Institute of Technology, Késmárk Utca 28/A, Budapest 1158, Hungary*

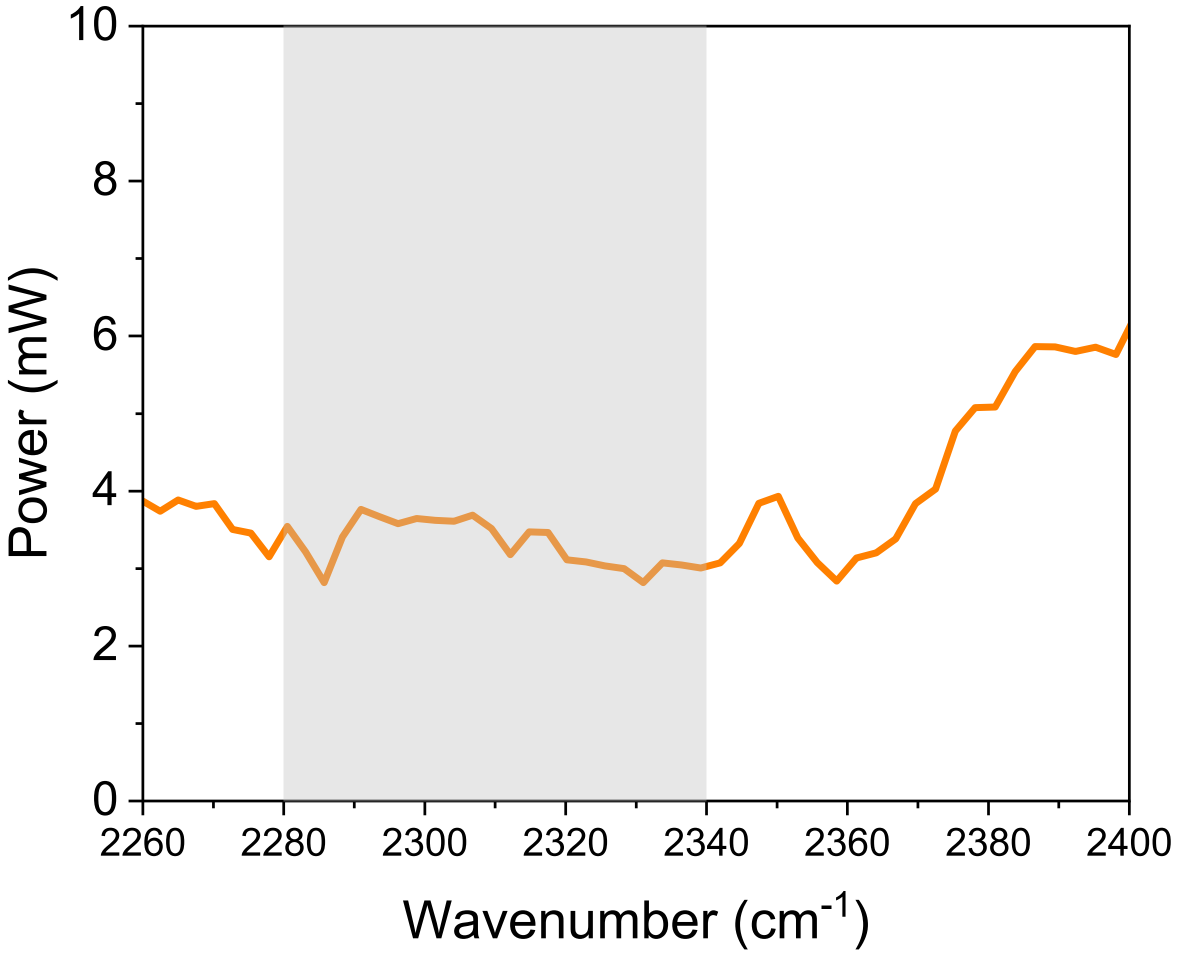

[4.] *Present address: Gedeon Richter Plc., Spectroscopic Research Department, 19-21 Gyömrői út, Budapest 1103, Hungary*

[5.]

Figure S1: The power curve of the laser employed, with the shaded gray area denoting the frequency range where the CO_2 stretch vibration was detected.

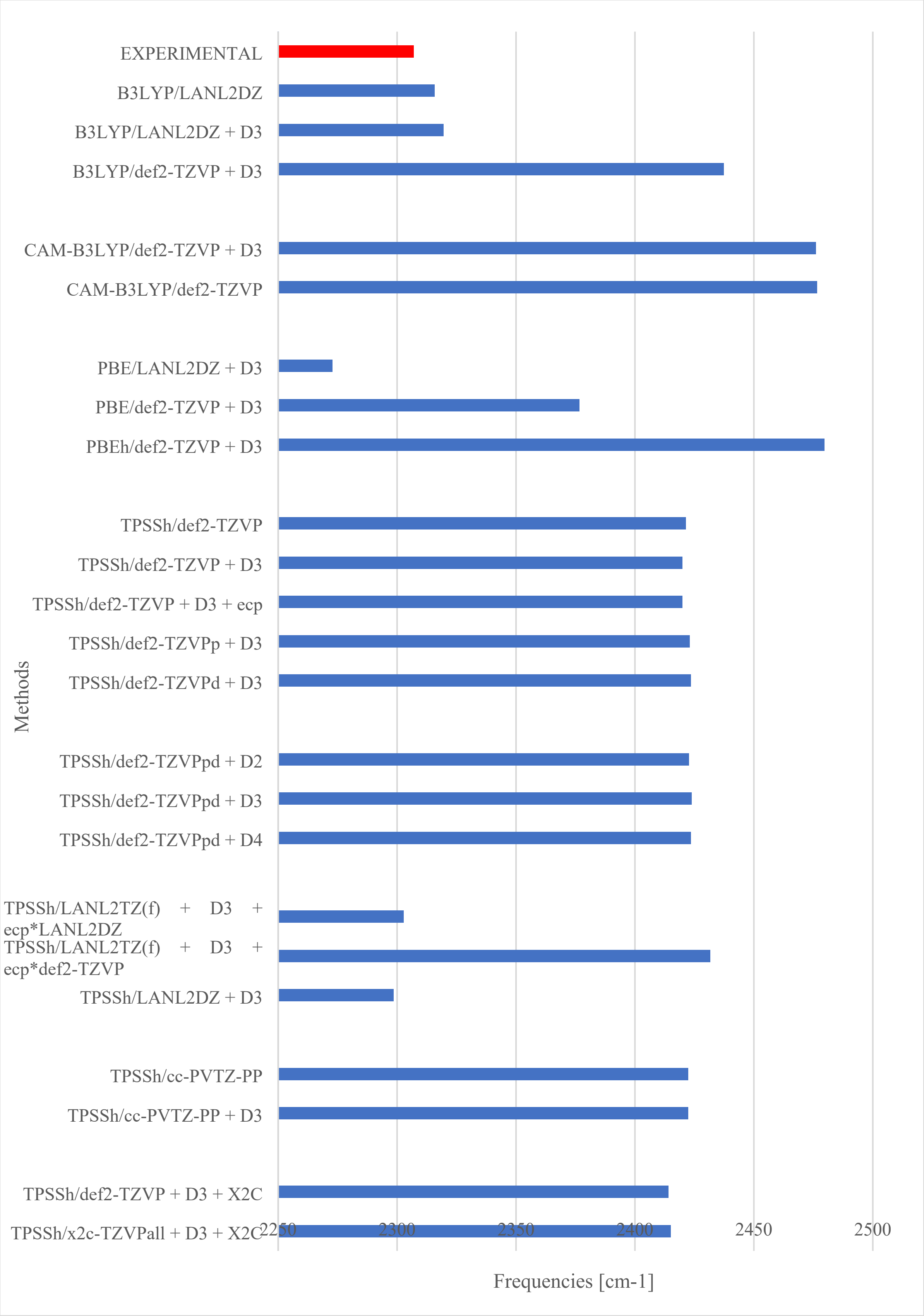

Figure S2: Benchmarking of different methods for harmonic frequency calculations on the asymmetrical stretching vibrational mode of the CO_2 in the $Cu_4[CO_2]^+$ adduct, with carbon mass corresponding to the natural abundance. Ecp*XXX the application of an effective core potential.

Method	TPSSh/LANL2DZ	TPSSh/def2-TZVP	TPSSh-D3/def2-TZVP	TPSSh/cc-pVTZ
**Harmonic**	2267	2400	2400	2406
**TOSH**	2218	2352	2351	2357
**VCI[1]**	2272	2408	2407	2413
**VCI[2]**	2226	2359	2359	2365
**VCI[3]**	2229	2362	2361	2367
**VCI[4]**	2220	2353	2352	2358
**VCI[5]**	2218	2352	2351	2357
**VCI[6]**	2218	2352	2351	2357

Table S1: Benchmarking of different methods for anharmonic calculations of the asymmetrical stretching vibrational mode of gas phase CO_2 molecule, with carbon mass set corresponding to natural abundance. The experimental value[1] is 2349 cm^{-1}.

TPSSh-D3/def2-TZVP

Method	$Cu[CO_2]^+$	$Cu_2[CO_2]^+$	$Cu_3[CO_2]^+$	$Cu_4[CO_2]^+$
**Experimental**	**2316**	**2323**	**2318**	**2312**
**Harmonic**	2429	2435	2431	2419
**TOSH**	2381	2385	2384	
**VCI[1]**	2434	2437	2437	
**VCI[2]**	2389	2392	2392	
**VCI[3]**	2391	2395	2394	
**VCI[4]**	2382	2386	2385	
**VCI[5]**	2381	2385	2384	
**VCI[6]**	2381	2385	2384	

Anharmonic scaling factor (n=1): 0.98
Anharmonic scaling factor (n=2): 0.98
Anharmonic scaling factor (n=3): 0.98

TPSSh/LANL2DZ

Method	$Cu[CO_2]^+$	$Cu_2[CO_2]^+$	$Cu_3[CO_2]^+$	$Cu_4[CO_2]^+$
**Experimental**	**2316**	**2323**	**2318**	**2312**
**Harmonic**	2325	2311	2307	2300
**TOSH**	2277	2262	2257	
**VCI[1]**	2326	2314	2309	
**VCI[2]**	2284	2270	2265	
**VCI[3]**	2287	2272	2268	
**VCI[4]**	2278	2263	2259	
**VCI[5]**	2277	2262	2258	
**VCI[6]**	2277	2262	2257	

Anharmonic scaling factor (n=1): 0.98
Anharmonic scaling factor (n=2): 0.98
Anharmonic scaling factor (n=3): 0.98

Table S2: Benchmarking of TPSSh-D3/def2-TZVP and TPSSh/LANL2DZ methods for anharmonic calculations of the asymmetrical stretching vibrational mode of $Cu_n[CO_2]^+$ complexes with n = 1-4, with carbon mass corresponding to natural abundance.

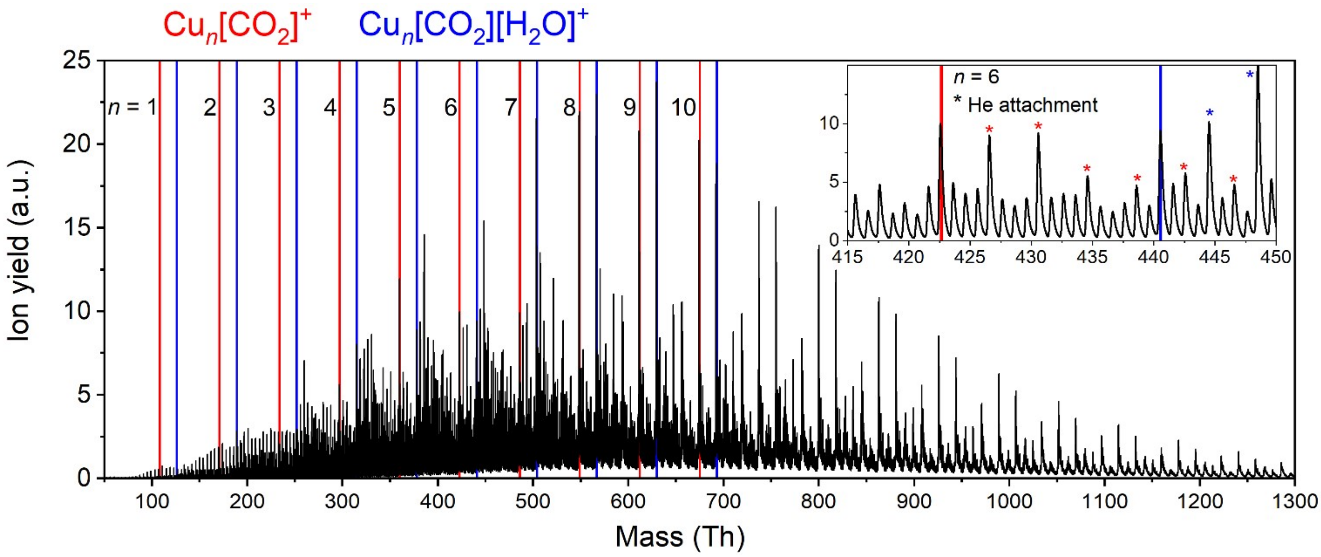

Figure S3: Mass spectrum where the experimental setup was optimised to create larger Cu_n clusters. The red and blue vertical lines highlight the positions of the $Cu_n[CO_2]^+$ and $Cu_n[CO_2][H_2O]^+$, respectively, for n = 1 – 10. In the inset the He attachment on the $Cu_6[CO_2]^+$ and $Cu_6[CO_2][H_2O]^+$ cluster is shown with red and blue asterisks accordingly.

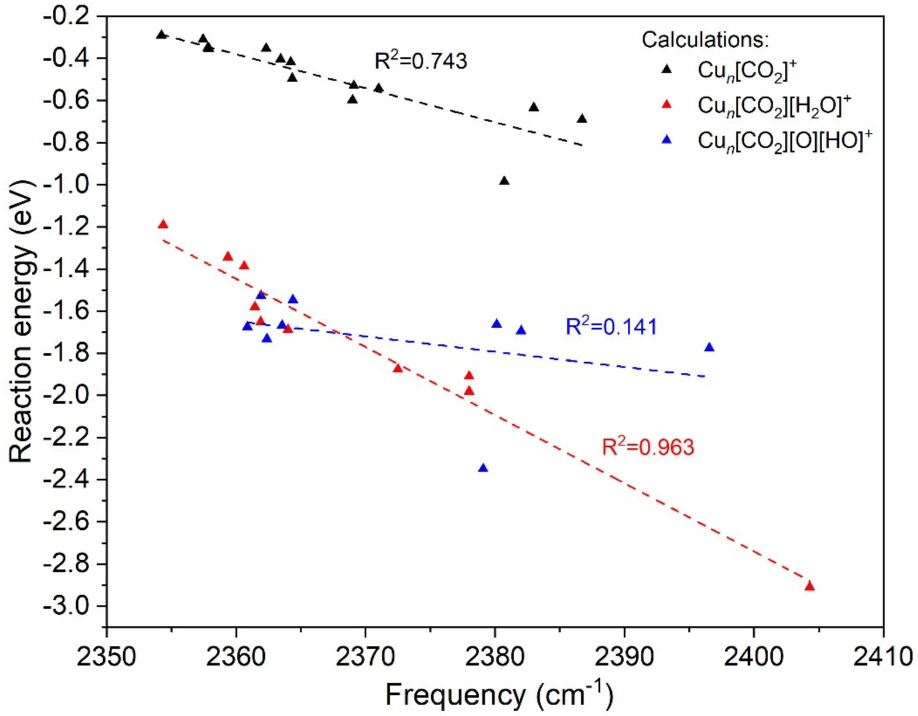

Figure S4: Correlation between binding energies and position of the asymmetric stretching vibrational mode of the bound CO_2 for all calculated Cu$_n$[CO$_2$]$^+$, Cu$_n$[CO$_2$][H$_2$O]$^+$ and Cu$_n$[CO$_2$][H][OH]$^+$ structures. The linear fit is shown with a dashed line and R^2 is calculated for each system. With carbon mass corresponding to natural abundance.

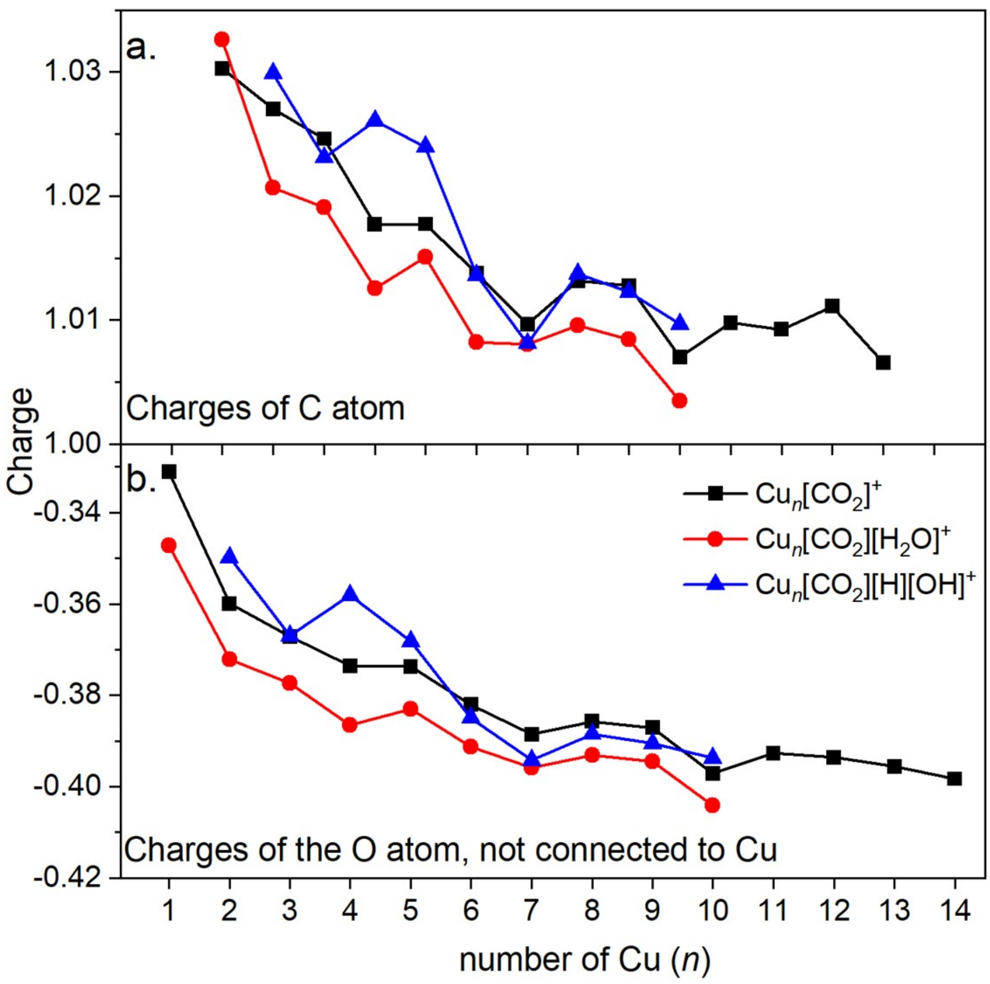

Figure S5: a. Charges of the C atom in the CO_2 as a function of different cluster-complex sizes. b. Charges of the O atom (which is NOT connected to the cluster) in the CO_2 as a function of different cluster-complex sizes.

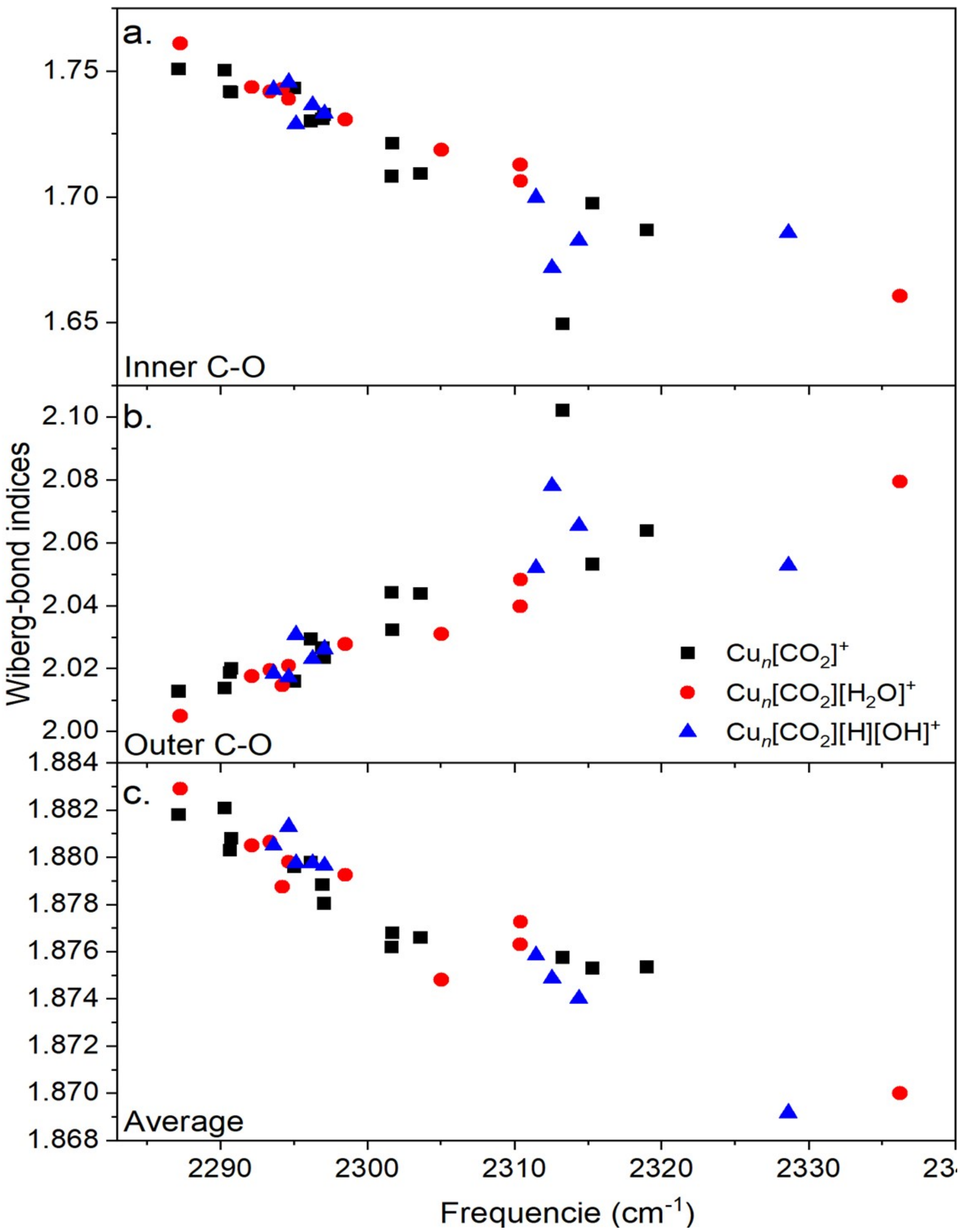

Figure S6: Wiberg bond indices of a. the C-O bond (where oxygen is connected to the cluster, i.e. inner oxygen); b. the C-O bond (where oxygen is not connected to the cluster, i.e. outer oxygen); c. both C-O bonds (inner and outer) with respect to the CO_2 asymmetric stretch vibrational frequency.

1. T. Shimanouchi, *Tables of molecular vibrational frequencies*, National Bureau of Standards Washington, DC, 1972.
